# A Novel De Novo Nonsense Pathogenic Variant in *IGSF1* Resulting in Central Hypothyroidism and Transient GH Deficiency

**DOI:** 10.1210/jcemcr/luaf033

**Published:** 2025-03-28

**Authors:** James Blackburn, Banupriya Sivasanker, Shahida Ahmed, Birgit van Meijgaarden, Marta Korbonits, Evelien Gevers

**Affiliations:** Department of Paediatric Endocrinology and Diabetes, Barts Health NHS Trust, Royal London Hospital, Whitechapel, London E1 1BB, UK; Centre for Endocrinology, Queen Mary University of London, London EC1M 6BQ, UK; Department of Paediatric Endocrinology and Diabetes, Barts Health NHS Trust, Royal London Hospital, Whitechapel, London E1 1BB, UK; Basildon University Hospital, Mid and South Essex NHS Foundation Trust, Nethermayne, Basildon, Essex SS16 5NL, UK; Basildon University Hospital, Mid and South Essex NHS Foundation Trust, Nethermayne, Basildon, Essex SS16 5NL, UK; Centre for Endocrinology, Queen Mary University of London, London EC1M 6BQ, UK; Department of Paediatric Endocrinology and Diabetes, Barts Health NHS Trust, Royal London Hospital, Whitechapel, London E1 1BB, UK; Centre for Endocrinology, Queen Mary University of London, London EC1M 6BQ, UK

**Keywords:** central hypothyroidism, macroorchidism, delayed puberty, hypogonadism, tall stature, obesity

## Abstract

The main features of immunoglobulin superfamily, member 1 (*IGSF1*) deficiency are central hypothyroidism and macroorchidism. The phenotype can be variable and may include macrosomia, hypoprolactinemia, growth hormone (GH) secretory abnormalities, delayed puberty, and obesity. We describe a novel de novo nonsense pathogenic variant c.3343C > T, p.(Gln1115*) that localizes to the 12th and last immunoglobulin-like loop in the C-terminal domain of the protein. The patient was born in breech with a birth weight of 3.7 kg. At presentation aged 15 years, he had obesity, central hypothyroidism, reduced attention, macroorchidism with delayed adrenarche and pubertal development. There was evidence of transient biochemical GH deficiency with normoprolactinemia and a small pituitary on magnetic resonance imaging, although interpretation of dynamic GH testing was difficult due to poor adherence with levothyroxine. He continued growing without GH treatment, and after spontaneous puberty, insulin-like growth factor-1 concentration was high. At age 22 years, he was 190 cm (+1.9 SDS), 155.4 kg (body mass index 43.05), with 35 mL testes. The transient GH deficiency and normoprolactinemia support a role for *IGSF1* in somatotroph function.

## Introduction

Central hypothyroidism (CeH) is a disorder of low free serum thyroxine caused by a lack of stimulation of a normal thyroid gland, either due to pituitary (secondary hypothyroidism) or hypothalamic dysfunction (tertiary hypothyroidism) [1]. Most commonly, central hypothyroidism is part of combined pituitary hormone deficiency. Isolated hypothyroidism accounts for 40% of CeH, with an incidence of 1:40 000 [2]. There are currently 5 genes associated with isolated CeH: *TSHB*, *TRHR*, *TBL1X*, *IRS4*, and *IGSF1* [3]. Variants in immunoglobulin superfamily, member 1 (*IGSF1*) are the most common genetic cause of central CeH [4] but may cause other pituitary hormone deficiencies.

*IGSF1* is highly expressed in thyrotrophs, somatotrophs, and the testes. *IGSF1* is located on Xq26.2 and inherited in an X-linked manner; therefore, IGSF1 deficiency primarily affects males. A minority of females with heterozygous *IGSF1* pathogenic variants have CeH, but with a milder phenotype [5]. Pathogenic variants in *IGSF1* were first described in patients with CeH who were noted to have macroorchidism [6, 7]. Evidence suggests reduced production of thyrotropin (TSH) is seen in patients with pathogenic variants in *IGSF1* [8]. Subsequently, the clinical features of patients with *IGSF1* pathogenic variants have expanded and include delayed puberty, hypoprolactinemia, transient partial growth hormone (GH) deficiency, and increased body mass index (BMI), with or without macroorchidism [6]. Somatotroph hyperfunction has been described in mice and humans with IGSF1 deficiency [9] and in line, IGSF1-deficient adult mice are heavier, larger, and skinnier than wild-type littermates [7]. Deficits in attention [10, 11], high birth weight, large head circumference, low dehydroepiandrosterone sulphate (DHEAS), and low cortisol concentrations and relatively high follicle-stimulating hormone/luteinizing hormone (FSH/LH) ratio have been described in humans [10].

The patient presented here carries a novel de novo nonsense pathogenic variant of *IGSF1* and has a clinical phenotype of above average birth weight, central hypothyroidism, delayed puberty, obesity, transient partial GH deficiency followed by high insulin-like growth factor-1 (IGF-1) concentration, macroorchidism, reduced attention, low DHEAS, and normoprolactinemia.

## Case Presentation

A 15-year-old boy was referred to a tertiary pediatric clinic for pubertal delay, obesity, and abnormal thyroid function tests (free thyroxine [FT4] 6.5 pmol/L (8.4-19.1 pmol/L) [0.51 ng/dL (0.65-1.48 ng/dL)], TSH 3.23 mIU/L [0.3-5.0 mIU/L]). The patient and family reported increasing food-seeking behavior for the past few years; his diet was carbohydrate rich, and he rarely exercised. He was born breech by cesarian delivery, weighing 3.7 kg (+0.32 SD score [SDS]). At age 2 years, weight was 11 kg (−1.38 SDS), height 85 cm (−0.75 SDS), and head circumference 52 cm (+2.30 SDS). He had normal early milestones (walking and talking) and cognition was reportedly normal although lack of attention was noted. He was recently diagnosed with metabolic dysfunction–associated steatotic liver disease (MASLD).

His mother is 168 cm (+0.70 SDS), had menarche aged 13 years and had type 2 diabetes from age 37 years. His father is 183 cm (+0.91 SDS) and reportedly weighed 115 to 145 kg and had normal puberty. Mid-parental height was 182 cm. The patient has one older brother with normal height and puberty.

At presentation, height was 166 cm (−0.52 SDS), weight 104.3 kg (+3.5 SDS), and BMI 38.5 (+3.45 SDS). Tanner staging was P1G1A1, with 8-mL testes. He had general obesity and moderately severe acanthosis nigricans.

## Diagnostic Assessment

Investigations at age 15 years confirmed central hypothyroidism, FT4 7.3 pmol/L (8.4-19.1 pmol/L) [0.57 ng/dL (0.65-1.48 ng/dL)], TSH 1.53 mIU/L (0.3-5.0 mIU/L), LH 0.7 IU/L, FSH 4.1 IU/L, testosterone 7.6 nmol/L [219 ng/dL), IGF-1 11.2 ng/mL (13.5-66 ng/mL) [1.47 nmol/L (1.77-8.65 nmol/L)], prolactin 136 IU/L (90-300 IU/L) [6.39 µg/L (4.23-14.1 µg/L)], antimüllerian hormone 28.1 pmol/L (5.5-103 pmol/L). A luteinizing hormone–releasing hormone (LHRH) test showed a peak LH 4.8 IU/L and FSH 12.8 IU/L. Short synacthen test and adrenocorticotropin (ACTH) were normal (peak cortisol 588 nmol/L [21.21 µg/dL] at 60 minutes, ACTH 3.69 pmol/L (<11 pmol/L) [18 ng/mL (<50 ng/mL)]). Glycated hemoglobin A_1c_ was 41 mmol/mol and oral glucose tolerance test (OGTT) showed fasting hyperglycemia (6.5 mmol/L [117.1 mg/dL]) but normal 120-minute glucose concentration.

**Figure 1.**
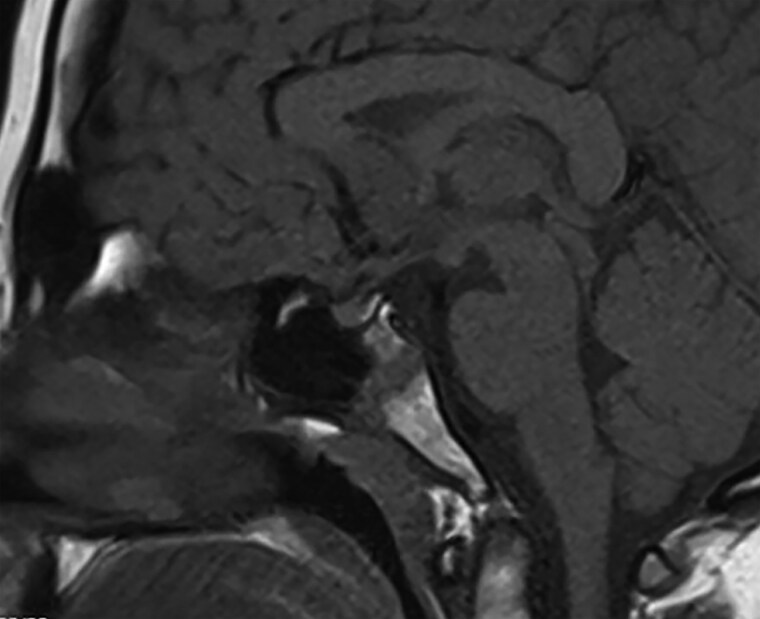
The patient had a small anterior pituitary gland. Magnetic resonance imaging of the head of the patient shows the anterior and posterior pituitary gland to be normally located. The anterior pituitary is small compared to expected parameters for age.

Bone age was 14 years, delayed by 1 year. Magnetic resonance imaging of the head (Fig. 1) showed a slightly small anterior pituitary gland for age with a normally positioned posterior pituitary. Ultrasound of the liver showed fatty infiltration with no focal lesions.

## Treatment

The patient commenced oral levothyroxine 25 µg daily, which was gradually increased to 175 µg. FT4 was frequently low due to poor adherence to medication. He was started on metformin treatment and given lifestyle advice to reduce calorie consumption and increase exercise.

## Outcome and Follow-up

Having commenced levothyroxine, an unprimed glucagon-stimulation test showed no response in GH (GH <0.1 µg/L at all time points), but FT4 was low (6.4 pmol/L [8.4-19.1 pmol/L] [0.50 ng/dL (0.65-1.48 ng/dL)]). Following the low GH response to glucagon-stimulation testing and after a reported improvement in medication compliance and normal FT4 concentration when booking, a gold-standard insulin tolerance test (ITT) was organized, with priming as he had pubertal delay and testes volume was unreliable due to the IGSF1 deficiency. ITT was performed at age 17.5 years due to frequent low FT4 and nonattendance. When the ITT was performed, he was growing fast. The GH peak was 4.21 µg/L (normal > 6.7 µg/L; Table 1), in line with GH deficiency. However, again the patient was noted to have low FT4 (6.8 pmol/L [10.5-24.5 pmol/L] [0.53 ng/dL (0.82-1.9 ng/dL)]) at the time of investigation. A month later the FT4 had improved to 9.9 pmol/L (0.77 ng/dL) and IGF-1 was above reference range at 73.1 nmol/L (16.9-63.9 nmol/L) [558 ng/mL (129-487.5 ng/mL)] with a high-normal insulin-like growth factor-binding protein 3 (IGFBP3) of 5.7 mg/L (normal 2.7-6.3 mg/L).

**Table 1.** Stilboestrol primed insulin tolerance test

Time, min	0	30	45	60	90	120
Glucose	4.6 mmol/L 82.8 mg/dL	2.0 mmol/L 36.0 mg/dL	2.8 mmol/L 50.5 mg/dL	3.5 mmol/L 63.1 mg/dL	4.0 mmol/L 72.1 mg/dL	4.3 mmol/L 77.5 mg/dL
Cortisol	211 nmol/L 7.65 µg/dL	169 nmol/L 6.13 µg/dL	564 nmol/L 20.4 µg/dL	662 nmol/L 24.0 µg/dL	622 nmol/L 22.55 µg/dL	538 nmol/L 19.5 µg/dL
GH	2.28 µg/L	1.29 µg/L	3.57 µg/L	4.21 µg/L	3.04 µg/L	2.20 µg/L

Abbreviation: GH, growth hormone.

Following levothyroxine treatment, he progressed through puberty and his macroorchidism increased rapidly with testicular volumes of 15 mL at age 16 years, 30 mL at age 17 years (P3G3A2), and 35 mL at age 18 years (P4G4A2). DNA sent for Sanger sequencing of *IGSF1* showed a novel nonsense pathogenic variant c.3343C > T, p.(Gln1115*), causing a premature stop-codon, classified as pathogenic using American College of Medical Genetics and Genomics guidelines [12]. The variant has not been previously described in population-based genetic databases (ClinVar and GnomAD). His parents did not carry the variant.

**Figure 2.**
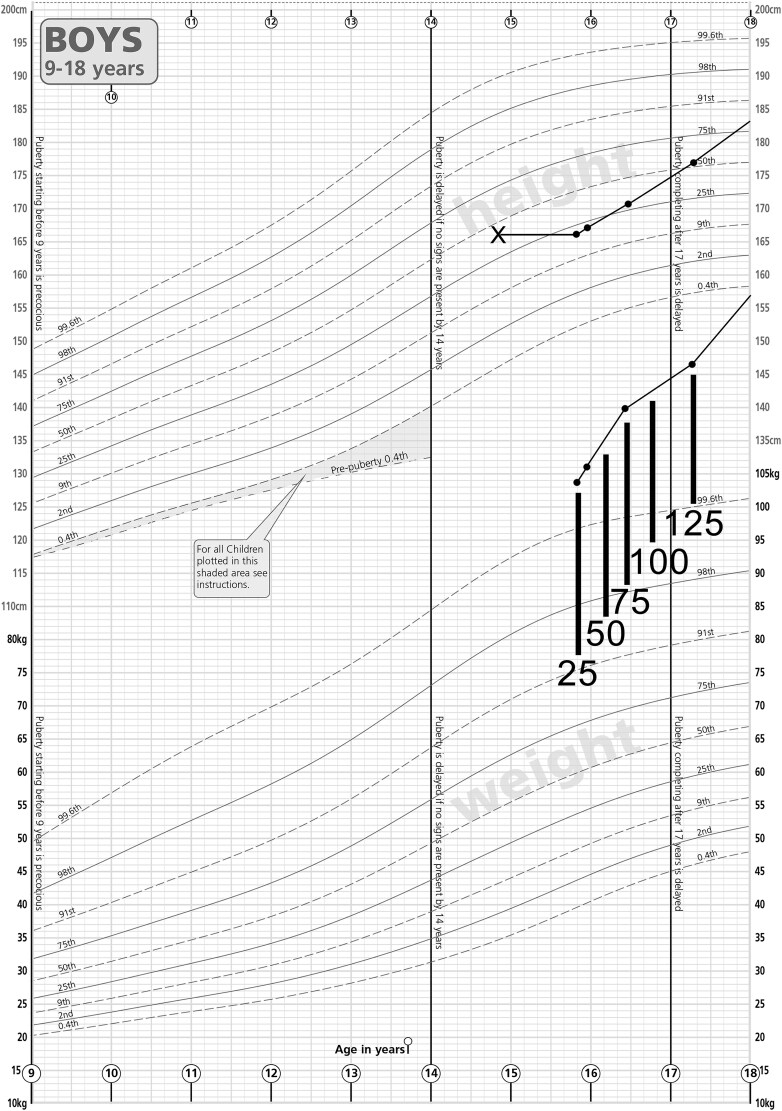
A gain in height percentile was observed after commencing levothyroxine. Growth chart showing height and weight measurements taken at various time points (single dots). The height percentile of the patient increases from the 25th percentile to greater than the 50th after commencing levothyroxine (dose in micrograms indicated by associated lines and numbers). Bone age (marked cross) was delayed by 1 year. The weight of the patient remains well above the 99th percentile throughout the course of his treatment.

His growth normalized with a delayed pubertal growth spurt that was ongoing at age 18 years (Fig. 2). At this time, inhibin B was 271 ng/L (25-325 ng/L), LH 2.2 IU/L, FSH 9.2 IU/L, and early-morning fasting testosterone 7.9 nmol/L (227.6 ng/dL). He reached adult height between age 20 and 22 years at 190 cm, in line with his father's height of 183 cm. His weight and BMI increased rapidly. DHEAS was low at age 21 years, (1.5 µmol/L [2.3-10 µmol/L] [55.6 µg/dL (85.2-370.4 µg/dL]).

Further investigation of the GH axis following progression through puberty showed variable IGF-1 without GH therapy, initially in the reference range (IGF-1 6.8 nmol/L (1.8-7.9 nmol/L) [52 ng/mL (14-61 ng/mL)]), IGFBP3 5.8 mg/L [3.1-7.9 mg/L]) and then above the reference range at age 21 years (49.9 nmol/L (14.8-41.9 nmol/L) [381 ng/mL (113-320 ng/mL)]). At the most recent clinical review (age 22 years), height was 190 cm, weight 155 kg, BMI 43.05 and, blood pressure 139/79 mm Hg. While prescribed 175 μg levothyroxine, FT4 was still low (8.7 pmol/L) (0.68 ng/dL) with TSH of 1.89 mU/L, likely indicating ongoing nonadherence. In view of the high IGF-1, an OGTT as a GH suppression test was performed to ensure the high IGF-1 concentration was not secondary to GH overproduction. OGTT was normal (GH < 0.03 µg/L) and IGF-1 subsequently normalized to 270 ng/mL (112-320 ng/mL) [35.4 nmol/L (14.7-41.9 nmol/L]). Gonadotrophins were within the reference range (LH 6.1 IU/L, FSH 5.5 IU/L) with a low-normal random testosterone concentration of 10.7 nmol/L (8.6-29 nmol/L) (308.4 ng/dL [247.8-835.7 ng/dL]). Serum albumin was 48 g/L [4.8 g/dL), with a sex hormone–binding globulin of 9 nmol/L (1.01 μg/mL). Calculated free testosterone was 0.327 nmol/L (9.42 ng/dL) (3.05%) and bioavailable testosterone 8.51 nmol/L (245.24 ng/dL [79.5%]). Laboratory reference ranges were not available for free testosterone; however, consensus guidelines state the reference range for free testosterone is greater than 0.25 nmol/L and treatment is suggested if free testosterone is below 0.18 nmol/L [13].

## Discussion

We describe a novel *IGSF1* variant c.3343C > T, p.(Gln1115*). To our knowledge this is the first time this variant and a “de novo” variant has been described as all cases described where parents were tested have an inherited *IGSF1* variant. In this case the parents were tested and did not carry the variant. The case demonstrates some common and less common features of the condition and further evidence of dysregulated GH secretion. The patient had high birth weight, large head circumference, obesity, central hypothyroidism, reduced attention, delayed puberty, macroorchidism, and some degree of GH deficiency followed by high IGF-1 concentrations, but no prolactin deficiency. LHRH testing showed a low LH peak, but adult FSH and LH were within reference ranges, with low-normal adult testosterone concentration, and normal inhibin B. FSH/LH ratio was high (4.2; reference range, 0.7-1.2 [10]) at age 18 years, but normalized afterward.

The novel *IGSF1* variant results in truncation in the last (12th) immunoglobulin-like loop in the C-terminal domain of the protein. Other pathogenic variants in this region have been described, mostly deletions and truncations [7]. Variants in this region impair normal trafficking of the IGSF1 protein, resulting in endoplasmic reticulum retention and absence of expression at the cell surface [14]. Nonsense-mediated decay may also occur. There is no genotype-phenotype correlation of pathogenic variants that affect the 12th immunoglobulin-like loop. Pathogenic variants predicted to affect other areas of the protein and complete or partial gene deletions have similar clinical features [7]. Indeed, clinical features of patients with the same *IGSF1* pathogenic variant are highly variable, suggesting other genes may influence the phenotype and penetrance may be variable. This has been demonstrated in siblings with the same pathogenic variant who had significantly different clinical presentations [15].

IGSF1 is highly expressed in the anterior pituitary of humans [16]. In thyrotrophs, IGSF1 deficiency leads directly to reduced TSH production [17] and indirectly to reduced TSH-releasing factor (TRH) receptor expression and reduced TRH signaling, thus resulting in reduced TSH production [18]. Nevertheless, the exact role of IGSF1 remains unclear. One of the less well studied and understood features of patients with *IGSF1* pathogenic variants are the effects on other cell lineages of the pituitary. IGSF1 is expressed in the gonadotrophs, lactotrophs, and somatotrophs in rodents [19, 20] and in the hypothalamus, but the role of IGSF1 in pituitary hormone secretion remains unclear.

The effect of IGSF1 on somatotrophs is suggested by several case reports showing GH dysregulation in children with *IGSF1* variants [6, 14, 21]. Delayed growth in this patient may be due to a combination of hypothyroidism, GH deficiency (potentially partially related to hypothyroidism), and a delayed pubertal growth spurt. Pubertal growth spurt is associated with increased IGF-1 concentration. Overproduction of GH was not proven in our patient and therefore the high IGF-1 is likely related to a late pubertal growth spurt, and indeed normalized afterward. Interestingly, mice with *Igsf1* deficiency are larger and have hypersecretion of GH from somatotrophs [9], and case series have shown adults can have higher IGF-1 concentrations and acromegalic features, with oversecretion of GH [9, 22]. In our patient, glucagon stimulation and primed ITTs suggested a degree of GH dysregulation initially, although caution is required as FT4 concentration at the time of GH testing was low due to nonadherence to treatment. Our patient developed high IGF-1 concentrations, mimicking the mouse model, although this normalized spontaneously and may be related to his delayed pubertal growth spurt, and overproduction of GH was not proven with an OGTT.

Recent case series have suggested that in addition to delayed testosterone increase, patients can develop hypogonadotropic hypogonadism [8]. There was no clear evidence of this in our patient as testosterone concentration was at the lower end of the reference range, and free testosterone was above the suggested treatment range [13]. High FSH or FSH/LH ratios have been described previously too [23], but the most recent FSH/LH ratio in our patient was normal.

Another recently identified feature of patients with *IGSF1* pathogenic variants [24] and demonstrated in this case is development of MASLD, likely due to obesity or suboptimally treated hypothyroidism [24].

In conclusion, the de novo novel nonsense pathogenic *IGSF1* variant c.3343C > T, p.(Gln1115*) results in a severe phenotype with many of the previously described features of IGSF1 deficiency, including dysregulated GH secretion, but no hypoprolactinemia, adding to our knowledge of IGSF1 deficiency syndrome and its variable phenotype.

## Learning Points

- Features of IGSF1 deficiency can be highly variable between patients and may change over time.
- IGSF1 pathogenic variants can affect somatotrophs and GH secretion.
- Most IGSF1 variants are inherited, and this is the first time a “de novo” variant is reported.

## Data Availability

Data sharing is not applicable to this article as no data sets were generated or analyzed during the present study.

## Abbreviations

ACTH: adrenocorticotropin
BMI: body mass index
CeH: central hypothyroidism
DHEAS: dehydroepiandrosterone sulphate
FSH: follicle-stimulating hormone
FT4: free thyroxine
GH: growth hormone
IGF-1: insulin-like growth factor-1
IGFBP3: insulin-like growth factor-binding protein 3
*IGSF1*: immunoglobulin superfamily, member 1
ITT: insulin tolerance test
LH: luteinizing hormone
LHRH: luteinizing hormone–releasing hormone
MASLD: metabolic dysfunction–associated steatotic liver disease
OGTT: oral glucose tolerance test
TRH: TSH-releasing factor
TSH: thyrotropin
